# The efficacy of pulsed-xenon ultraviolet light technology on Candida auris

**DOI:** 10.1186/s12879-019-4137-6

**Published:** 2019-06-19

**Authors:** Caroline Maslo, Moira du Plooy, Jennifer Coetzee

**Affiliations:** 1 Department of Quality Leadership, Netcare Hospitals, Johannesburg, South Africa; 2 Mycology Unit, Ampath National Reference Laboratory, Centurion, South Africa; 3 Department of Clinical Microbiology and Molecular Biology, Ampath National Reference Laboratory, Centurion, South Africa

**Keywords:** Candida spp., Pulsed-xenon ultraviolet light, Disinfection

## Abstract

**Background:**

*Candida auris *is an emerging, often multi-resistant, yeast that causes invasive infections in healthcare settings. Patients may be colonized for months and *C. auris *has been shown to remain viable on surfaces for at least 14 days. It is widely considered that the environment may be a reservoir for transmission of *C. auris*. The efficacy of pulsed-xenon ultraviolet (PX-UV) mobile devices on *C. auris *has not been tested previously. In a laboratory setting, we tested efficacy of a PX-UV system on *C. auris *and *C. parapsilosis*, another candida known to be responsible for outbreaks in healthcare settings and survive for at least 28 days in the environment.

**Methods:**

Cultures and growth of clinical strains of *C. parapsilosis* and *C. auris *was carried out in a broth liquid culture medium at 37 °C until concentration ranges 10 ^5^–10 ^6^ colony-forming units (CFUs) per millilitre were obtained. Glass slides were inoculated with 10 μl of *C. auris *stock culture and allowed to dry. Slides were positioned perpendicular to the floor at a distance of 1.25 m from the floor. Exposure time were run uninterrupted for 5-, 10- and 15-min cycles at 1- and 2-m distance.

**Results:**

There was a 99.4% reduction in *C. auris *CFU after a 5-min cycle at 1-m distance, and 99.6% reduction after a 10-min cycle at 2-m distance. There was a 98.5% reduction in *C. parapsilosis* CFU after a 5-min cycle at 1-m distance, and 95.2% reduction after a 10-min cycle at 2-m distance.

**Conclusions:**

The PX-UV mobile device is easy to use and has short cycle times that makes it easier to disinfect all areas outside the room where the patient received care. Further studies are needed in hospital environment, to assess the cumulative impact of repeated sessions.

## Background

*Candida auris* is an emerging, often multi-resistant, yeast that causes invasive infections in healthcare settings [1, 2]. *C. auris* has the ability to cause large healthcare outbreaks [3, 4]. Patients may be colonized for months [3, 5] and *C. auris* has been shown to remain viable on surfaces for at least 14 days [6, 7]. It is widely considered that the environment may be a reservoir for transmission of *C. auris*. The Centre for Disease Control and prevention (CDC) recommends post discharge terminal cleaning and disinfection of patients’ rooms and cleaning and disinfection of areas outside the rooms where patients received care [8]. It has been demonstrated that conventional cleaning and disinfection often lacks consistency and that additional disinfection, using non-touch technologies such as hydrogen peroxide vaporisation or germicidal ultraviolet (UV-C) light can further reduce the surface bioburden and transmission of microorganisms [9]. However, a recent publication by Cadnum and colleagues showed a relative resistance of *C. auris* and other candida species to UV-C and that extended exposure time (20 to 30 min) might be needed [10]. In their experiment, Cadnum and colleagues (2018) used a mobile device that emits 254-nm continuous UV-C light. We tested the hypothesis that the pulsed-xenon ultraviolet (PX-UV) system that emits broad spectrum UV-C (200–280-nm) in short pulses could reduce the exposure time needed to decrease *C. auris*. As a comparison, we also tested the PX-UV system on *C. parapsilosis*, another candida known to be responsible for outbreaks in healthcare settings and survive for at least 28 days in the environment [7].

## Methods

Prior cultures and growth of clinical strains of *C. parapsilosis* and *C. auris* was carried out in a broth liquid culture medium at 37 °C until concentration ranges 10^5^–10^6^ colony-forming units (CFUs) per millilitre were obtained. Glass slides were inoculated with 10 μl of *C. auris* stock culture and allowed to dry.

Slides were positioned perpendicular to the floor at a distance of 1.25 m from the floor. Exposure time were run uninterrupted for 5-, 10- and 15-min cycles. The experiment was conducted at 1-m distance from the centre of the robot and repeated at a 2-m distance. Tests were conducted in triplicate. Three controls treated in identical conditions were prepared with each organisms. The controls remained covered during the experiment. After exposure, the control and PX-UV exposed slides were incubated in 10 ml phosphate-buffered saline (PBS) and agitated for 30 s to extract CFUs. Serial dilutions were plated onto Chromagar Candida and incubated at 36*for 48 h. The total viable count was quantified into number of colony forming unit. The percent reduction in the pathogens were calculated in comparison with unexposed control slides. The experiment was repeated for *C. parapsilosis*.

## Results

The laboratory testing results for *C. auris* are detailed in Table 1, stratified according to cycle time (5, 10 and 15 min) and distance from the centre of the UV light bulb (1 and 2 m). At 1-m distance from the PX-UV light bulb, there was a reduction of 99.4% of *C. auris* CFU observed after a 5-min cycle, while no growth was observed at 10 and 15 min. At 2-m distance from the light bulb, the *C. auris* CFU reduction was 90.2% after a five minutes cycle, and 99.6% after a 10-min cycle. There was no growth at 15 min.

**Table 1:** Laboratory testing results for *C. auris* and *C. parapsilosis*

*C.auris*	Slide	1-Meter Distance^a^	2-Meter Distance^a^
Cycle Time	Control	185 CFU	185 CFU
5 min	Slide 1	1 (99.5%)	17 (90.8%)
	Slide 2	2 (98.9%)	22 (88.1%)
	Slide 3	No growth	15 (91.9%)
10 min	Slide 1	No growth	No growth
	Slide 2	No growth	2 (98.9%)
	Slide 3	No growth	No growth
15 min	Slide 1	No growth	No growth
	Slide 2	No growth	No growth
	Slide 3	No growth	No growth
*C. parapsilosis*	Slide	1-Meter Distance^a^	2-Meter Distance^a^
Cycle Time	Controls	91 CFU	185 CFU
5 min	1	No growth	74 (18.7%)
	2	1 (98.9%)	73 (19.8%)
	3	3 (96.7%)	83 (8.8%)
10 min	1	No growth	7 (92.3%)
	2	No growth	3 (96.7%)
	3	No growth	3 (96.7%)
15 min	1	No growth	No growth
	2	No growth	No growth
	3	No growth	No growth

^a^ Number of CFU (percent reduction compared to controls)

At 1-m distance, the average reduction in *C. parapsilosis* CFU was 98.5% after a 5-min cycle. There was no growth at 10 and 15 min. At 2-m distance, the average efficacy was only 15.7% after a 5-min cycle while it was 95.2% after a 10-min cycle. There was no growth after a 15-min cycle.

## Discussion

We found that at 1-m distance from the PX-UV light bulb, there was a 99.4% reduction in *C. auris* after a 5-min cycle and no growth after 10- and 15-min cycles. Similarly, for *C. parapsilosis* there was a 98.5% reduction after a 5-min cycle and no growth after 10- and 15- min cycles. We found that at 2-m distance, there was a 15.7% reduction in C. auris after a 5-min cycle, a 95.2% reduction after a 10-min cycle, and no growth after a 15-min cycle. For *C. parapsilosis*, there was a 90.2% reduction after a 5-min cycle, a 99.6% reduction after a 10-min cycle, and no growth after a 15-min cycle.

The limitations of this study include a relative small study numbers and only one strain of *C. auris*. The strengths of this study include the inclusion of two common Candida species with varying distances from the PX-UV bulb. Previous studies have not tested the efficacy of PX-UV for Candida spp. Further studies are needed in hospital environment, to assess the cumulative impact of repeated sessions.

*Candida auris* and *Candida parapsilosis* are two pathogens commonly involved in outbreaks in healthcare settings outbreaks. They are often recovered from the hospital environment, where they can survive for a long time. Additional disinfection with hydrogen peroxide vapour is effective on *C. auris* and *C. parapsilosis*. However, this method is labour intensive as it requires sealing of the bed space, or doors and vents in case of a single-bed room, and monitoring of the concentration of H_2_O_2_ inside the enclosure before permitting patients or staff to enter into the room. The cycle time can last 2 to 8 h according to the H_2_O_2_ vapour system used. By comparison, the PX-UV technology, is easy to use and has significantly shorter cycle times that makes it easier to disinfect all areas outside the room where the patient received care, as recommended by the CDC.

## Conclusions

We found a 99.6% reduction after a 10-min cycle at 2-m distance, and a 95.2% reduction after a 10-min cycle at 2-m distance. The PX-UV mobile device is easy to use and has short cycle times compared to other no-touch room disinfection technologies. Further studies are needed in hospital environment, to assess the cumulative impact of repeated sessions.

## Data Availability

The datasets used and/or analysed during the current study are available from the corresponding author on reasonable request.

## Abbreviations

C. auris: Candida auris
*C. parapsilosis*: *Candida parapsilosis*
CDC: Centre for Disease Control and prevention
CFUs: Colony-forming units
PBS: Phosphate-buffered saline
PX-UV: Pulsed-xenon ultraviolet
UV-C: Germicidal ultraviolet Light- C
